# Large language model based mutations in genetic improvement

**DOI:** 10.1007/s10515-024-00473-6

**Published:** 2025-01-21

**Authors:** Alexander E. I. Brownlee, James Callan, Karine Even-Mendoza, Alina Geiger, Carol Hanna, Justyna Petke, Federica Sarro, Dominik Sobania

**Affiliations:** 1 University of Stirling, Scotland, UK; 2 University College London, England, UK; 3 King’s College London, England, UK; 4 Johannes Gutenberg University Mainz, Mainz, Germany

**Keywords:** Large language models, Genetic improvement

## Abstract

Ever since the first large language models (LLMs) have become available, both academics and practitioners have used them to aid software engineering tasks. However, little research as yet has been done in combining search-based software engineering (SBSE) and LLMs. In this paper, we evaluate the use of LLMs as mutation operators for genetic improvement (GI), an SBSE approach, to improve the GI search process. In a preliminary work, we explored the feasibility of combining the *Gin* Java GI toolkit with OpenAI LLMs in order to generate an edit for the JCodec tool. Here we extend this investigation involving three LLMs and three types of prompt, and five real-world software projects. We sample the edits at random, as well as using local search. We also conducted a qualitative analysis to understand why LLM-generated code edits break as part of our evaluation. Our results show that, compared with conventional statement GI edits, LLMs produce fewer unique edits, but these compile and pass tests more often, with the OpenAI model finding test-passing edits 77% of the time. The OpenAI and Mistral LLMs are roughly equal in finding the best run-time improvements. Simpler prompts are more successful than those providing more context and examples. The qualitative analysis reveals a wide variety of areas where LLMs typically fail to produce valid edits commonly including inconsistent formatting, generating non-Java syntax, or refusing to provide a solution.

## Introduction

With the ever-growing size and complexity of software systems, their maintenance requires a huge amount of manual effort (Böhme et al. 2017). Therefore, methods for automating software maintenance and optimisation have been proposed. Although a lot of attention has been devoted to automated bug fixing, more and more techniques for improvement of non-functional properties of software such as runtime or memory consumption have emerged (Sarro 2023; Blot and Petke 2022; Hort et al. 2021).

Genetic Improvement (GI) (Petke et al. 2018) applies search-based techniques to improve such properties of existing software. Although GI has had success in the industry (Kirbas et al. 2021; Marginean et al. 2019), it remains limited by the set of mutation operators it employs in the search (Petke et al. 2023). Given that Large Language Models (LLMs) have found a wide range of applications in software engineering, in our preliminary work (Brownlee et al. 2024a) we checked if they could be used to generate new mutations for GI.

LLMs are able to process textual queries without additional training for the particular task at hand, and have been pre-trained on billion lines of code (Chen et al. 2021). Their use for software engineering tasks has had great success (Hou et al. 2023; Fan et al. 2023), showing promise also for program repair (Sobania et al. 2023; Xia and Zhang 2023). Kang and Yoo (2023) have suggested that there is untapped potential in using LLMs to enhance GI. GI uses the same mutation operators for different optimisation tasks. These operators are hand-crafted prior to starting the search and thus result in a limited search space.

In our preliminary work, we equipped a Genetic Improvement (GI) search-based framework called *Gin* (Brownlee et al. 2019) with a novel mutation operator that queried OpenAI’s API for generating a patch. Our initial results showed that mutations generated by LLMs can lead to viable program variants.

In this paper, we extend that work by investigating the effectiveness of using three different LLMs and three specialised prompts with *Gin* on five different open-source software projects. In particular, we make the following contributions:

1. We sample random mutations for each combination of LLMs, prompts, and software projects, and benchmark the performance against ‘statement’ edits commonly used in the GI community that copy, delete, replace, or swap elements of the AST (Petke et al. 2023). Our results show that, compared with conventional statement GI edits, LLMs produce fewer unique edits, but these compile and pass tests more often. OpenAI performed best over all target projects, finding test-passing edits an average of 77% of the time. The figures for local models Mistral and TinyDolphin were 31% and 7% compared to conventional statement edits at 12% on average. Simpler prompts were also more successful than those providing more context and examples.
2. We run local search to find runtime improvements. Our results show that OpenAI and Mistral were roughly equal in finding the best run-time improvements; Mistral found the maximum and highest average run time improvement on three projects, with OpenAI finding the best on the other two. TinyDolphin was able to find the highest average improvement on one project, JCodec .
3. We perform a qualitative analysis of the suggested edits from the LLMs. Our results show that valid edits were not found for a variety of reasons: from inconsistent formatting of code, or use of languages other than Java, in the response, to simply refusing to provide the requested suggestions. In particular, a provision of example changes in the prompt tended to lead to echoing of the examples in the LLM’s response, or erroneous responses.

In order to facilitate replication, reproduction and extension of our work, we make available the code, LLMs prompts, data and results at (Brownlee et al. 2024).

The rest of this paper is divided as follows: Sect. 2 describes our approach for incorporating LLM-generated mutations within *Gin* , a GI framework; Sect. 3 presents our research questions; Sect. 4 explains the experimental setup to evaluate our proposed approach with different LLMs and prompts; Sect. 5 presents our results; with Sect. 6 discussing threats to validity; Sect. 7 positions our work within existing literature; while Sect. 8 concludes our paper.

## Approach

We are investigating the application of LLMs to generate edits (code variants) for genetic improvement. In this section, we set out the essential preliminaries of Genetic Improvement (GI), edits, and testing (Subsection 2.1), and then move on to describe our approach to integrating an LLM within GI.

### Genetic improvement framework

Our test framework used the *Gin* (Brownlee et al. 2019) toolkit for Genetic Improvement in Java. *Gin* ’s approach to Genetic Improvement centres on the concept of *edits*: small changes to the code. Collections of edits, referred to as *patches*, are applied to a target application, compiled, and run using unit tests. The search for patches that make an improvement of some kind is carried out by a heuristic such as local search. The framework implemented in *Gin* is as follows:

**Profiling.** For each program, *Gin* ’s profiler determines a set of *hot* methods. In *Gin* , these are the methods seen most often at the top of the stack, when the full test suite is executed. The assumption is that these are the methods that consume most of the given program’s computational time. All edits are targeted at one of the *hot* methods identified by the profiling stage. Gin’s profiler also identifies the unit tests associated with each *hot* method by logging the calling test whenever a *hot* method is identified (Watkinson and Brownlee 2024).

**Applying Edits.** *Gin* uses JavaParser^1^ to parse a target program into an abstract syntax tree (AST). Edits are then made to the code by manipulating nodes in this tree. In our study, we have limited edits to only manipulating Java block statements; i.e. code enclosed in braces such as loop or conditional statement bodies, up to the level of method bodies. For the edits within a given patch, *Gin* applies the change to the target source code using JavaParser. After all patches are applied, it attempts to compile the modified code. If this is successful, we run the unit tests associated with the method that has been modified.^2^ In our experiments, this process was run for each patch in a freshly instantiated Java Virtual Machine (JVM) (i.e. *Gin* runs in one JVM, and creates a fresh second JVM for each patch to compile and run the target application). Upon completion, the outcome of each stage is recorded; this is summarised in our results as:

- *Valid* patches resulted in valid Java syntax that could be parsed by JavaParser and successfully inserted into the target program
- *Compiled* patches successfully passed compilation
- *Passed* patches passed all associated unit tests
- *Wall-clock time* the time as measured by Java’s System.nanoTime function
- *CPU time* the CPU time as measured by a call to Java’s ThreadMXBean.getThreadCPUTime() function

**Search.** We experimented with two types of search implemented within *Gin* : random search and local search. Random search is intended to provide some insight into the global search space. It simply generates patches each comprising a single edit selected uniformly at random among the space of all possible edits for the target program’s *hot* methods. Local search (Algorithm 1) is a random mutation hillclimber, starting with the working code and only accepting edits that offer a run time improvement while still passing the tests. The hillclimber is repeated for each of the top ten *hot* methods (those identified by the profiler as consuming the most CPU time). *neighbour()* generates a neighbour of an existing patch. With a 50% probability either an edit will be removed from the patch, or a new edit generated uniformly at random will be added.

**Algorithm 1:**
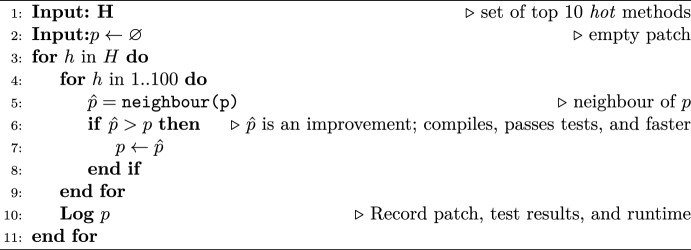
Upper level stochastic local search

### LLM edits

We utilise two LLM APIs to integrate LLM models into the GI toolbox *Gin* (Brownlee et al. 2019) to enable LLM edits within a GI framework: Langchain4J^3^ to access the OpenAI API and Ollama4j^4^ to access TinyDolphin and Mistral using the *Ollama* toolkit. *Gin* gets as input the name of the LLM to load and a prompt template to use. Both are fixed throughout a single experimental run. The specific LLM versions used in our evaluation are detailed in Sect. 4.

LLM edits are applied by selecting a block statement uniformly at random in the target *hot* method. This block’s content is $\texttt {<CODE>}$ in the prompt examples in the following section. The LLM is called with the specified prompt. The code blocks in the LLM response are identified using a regular expression ```(?:java)(.*?)```, which seeks text marked up as code either with or without the tag ‘java’. *Gin* uses JavaParser internally to represent target source files, so we attempt to parse each of the LLM suggestions with JavaParser. The first suggestion that JavaParser is able to parse then replaces the original block to generate the variant code.

### Prompt templates

**Fig. 1:**
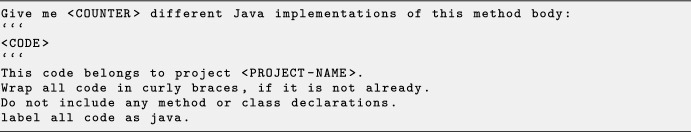
The basic prompt for LLM requests, with line breaks added for readability. Parameter placeholders are indicated by uppercase letters surrounded by angled brackets

In our previous work (Brownlee et al. 2024a), we experimented with three different prompts for sending requests to the LLM for both types of search: a simple prompt, a medium prompt, and a detailed prompt. All prompts requested 5 different Java implementations of a given code snippet, to increase the chance that one of the suggestions could be parsed. We found that the medium prompt (shown in Fig. 1) struck the right balance by being informative enough to yield valid code while not being overly aggressive in the edits, achieving a higher rate of compilable patches. The medium prompt provides information about the programming language, the project to which the code belongs, as well as formatting instructions.

**Fig. 2:**
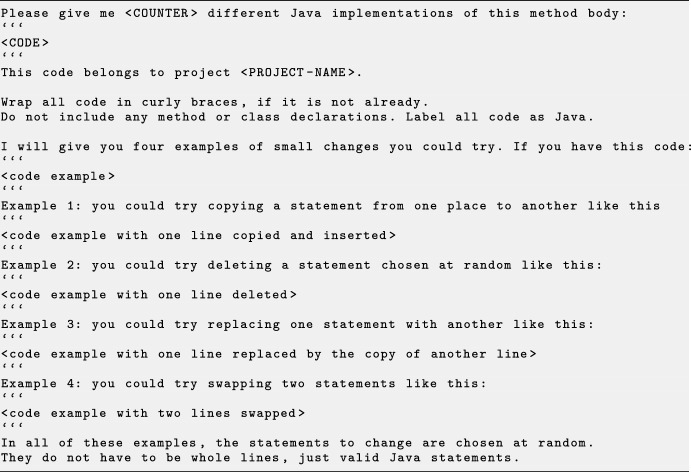
The small changes prompt for LLM requests, with line breaks added for readability. Parameter placeholders are indicated by uppercase letters surrounded by angled brackets

**Fig. 3:**
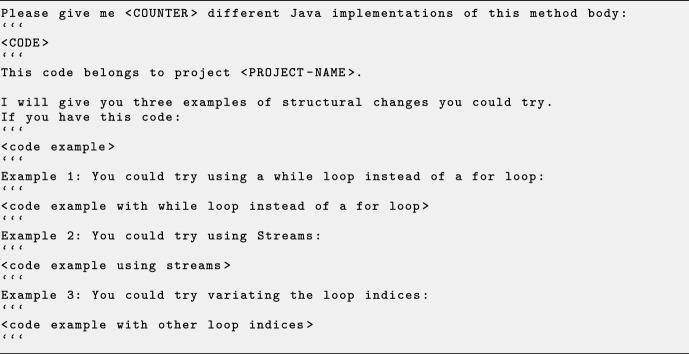
The structural changes prompt for LLM requests, with line breaks added for readability. Parameter placeholders are indicated by uppercase letters surrounded by angled brackets

We have implemented a new mechanism for fully parameterised prompt templates to explore further the efficiency of various prompts. *Gin* can receive a prompt template and the name of an LLM model to query. Each template includes placeholders for parameters (indicated by capital letters). These parameters are the number of different variations of the code at hand, the code itself, and the project name, all integrated into the template structure. We parameterised the medium prompt to create a template, which we denote as basic template (Fig. 1). We create two additional prompt templates that extend the basic template by examples: (1) small changes template suggesting, by examples, small GI edits (e.g. swap, remove, duplicate) as exemplified in Fig. 2, and (2) structural changes template suggesting examples related to structure (e.g. replace for loop in while loop) as shown in example Fig. 3. The examples provided in the small changes prompt and the structural changes prompt are fixed. In Sect. 5, we evaluate *Gin* using all three prompts, setting $\texttt {<COUNTER>}$ to five, thereby requesting five distinct variations of the code in question.

## Research questions

We aim to understand how often LLMs produce valid and effective program variants and how the LLM-generated mutations compare against traditional GI mutation operators. Therefore, our primary research question is as follows:

**RQ1:** How effective are LLMs at producing valid, compiling, and test passing program variants when prompted to produce an alternative implementation of a given method compared to traditional GI mutation operators?

We seek to understand further the impact of employing different LLMs, which can differ in size and may operate locally or in a server environment. It is worth noting that if smaller and local LLMs are sufficient, this could facilitate a broader uptake of the approach. Considering this, we ask:

**RQ2:** What is the difference in terms of efficacy between LLMs at producing program variants when prompted to produce alternative method implementations?

We explore the effect of using different prompts. We aim to identify essential information necessary for efficient prompting in the context of program variants, including assessing the potential benefits of using code examples to describe possible code changes. Thus, we ask:

**RQ3:** Which prompts are most effective in generating program variants produced by an LLM when prompted to produce alternative method implementations?

Moreover, we investigate if the generated variants not only pass the tests, but also improve upon the software property of interest, in our case - execution time.

**RQ4:** How effective are LLMs at producing runtime-improving software variants when prompted for alternative method implementations compared to traditional GI mutation operators?

Finally, we study the additional time overhead produced by LLM generated edits. Here, we ask:

**RQ5:** What is the difference in terms of efficiency between LLMs at producing program variants when prompted to produce alternative method implementations?

## Experimental setup

In this section we describe the experimental setup we followed to answer our research questions.

### Profiling

Gin’s profiler makes use of Java Flight Recorder, and works by sampling the call stack during a run of the test suite. In the following, we summarise the main aspects of this profiler, while a detailed description can be found in previous work (Watkinson and Brownlee, 2024).

The profiler starts at the top of the stack and works down until it finds the first appearance of a method in the target project (rather than Java API or library code) at 10ms intervals. A counter for each method is incremented each time it is found during profiling; the calling unit test is also logged. At the end of profiling, all methods are ranked in order of appearances during profiling. We could take the top-ranked methods from this ranking as the *hot* methods to be targeted, but this leads to different methods being identified as *hot* in each run. A small number of methods tend to be consistently identified as *hot*, with a long tail of less-*hot* methods appearing rarely , i.e., not being ranked at all. Given this context, *hot* methods to be targeted by the edits were identified using *Gin* ’s profiler tool by repeating the profiling 20 times and taking the intersection of the resulting 20 sets of methods assigned a ranking. Across the repeat runs, we identified the unit tests that had called each *hot* method during each repeat run and took the union of the unit tests for each *hot* method.

### Target projects

In our preliminary work, the target project for improvements in our experiments was the popular JCodec project. In this work, we extended our experiments to four additional projects, namely JUnit4 , Gson , Commons-Net and Karate . These additional projects were sourced by looking at popular projects rated with high stars, which use Java 17, employ Maven or Gradle as the build system, are open-source, and have a permissive licence. The project selection criteria were similar to those used in two previous studies with *Gin* (Petke et al. 2023; Watkinson and Brownlee 2024). From here, we tried to compile the project and run the *Gin* ’s EmptyPatchTester to ensure the project was compatible. Projects that successfully compiled, and were compatible with *Gin* , were selected to be included in our evaluation.

Table 1 presents all five projects with their URL and the branch checked out for the experiments as well as the number of Java files and Java code lines (denoted as SLOC). The size of the selected projects ranges from 226 Java files for Gson to 1182 files for JCodec . The SLOC range from 24787 for Commons-Net to 132538 for JCodec .

**Table 1:** Target projects with URLs and the checked-out versions as well as the number of Java files and Java code lines (denoted as SLOC)

Project	URL & branch	Java files	SLOC (Java)
JCodec	github.com/jcodec/jcodec (master, 7e52834)	1182	132538
JUnit4	github.com/junit-team/junit4 (r4.13.2)	471	31242
Gson	github.com/google/gson (gson-parent$-$2.10.1)	226	31011
Commons-Net	github.com/apache/commons-net (rel/commons-net$-$3.10.0)	281	24787
Karate	github.com/karatelabs/karate (v1.4.1)	488	44869

### Algorithm parameters

For the random sampling experiments, we set up the runs with statement-level edits (copy/delete/replace/swap from Petke et al. (2023)) and LLM edits, generating 1000 of each type at random. Line and statement level edits are the most commonly applied in GI (Petke et al., 2019): here we focus on statements as these have been shown to be more likely to lead to compiling and test-passing variants (Petke et al., 2023). A timeout of 10000 milliseconds was used for each unit test to catch infinite loops introduced by edits; exceeding the timeout counts as a test failure. For local search, experiments were set up similarly. There were 10 repeat runs (one for each of the top 10 hot methods) but the runs were limited to 100 evaluations resulting in 1000 evaluations in total, matching the random sampling. In practice, this amounted to 99 edits per run as the first was used to time the original unpatched code.

### LLM

We utilised Langchain4J version 0.18.0 and Ollama4j version 1.0.44 to integrate OpenAI and *Ollama* models, respectively. LLM prompts using the OpenAI API via the Langchain4J library were set with a temperature of 0.7, calling GPT 3.5 Turbo (specifically gpt $-$3.5-turbo-1106) ran directly on GPUs on servers in data centres managed by OpenAI . We employed *Ollama* to prompt from TinyDolphin (0f9dd11f824c, 637MB) and Mistral (61e88e884507, 4.1GB).

#### A note on the selection of language models

Between January 8th and 9th, 2024, we evaluated several language models. We tested their ability to write Java code and assessed the relevance of their outputs given a sample prompt for optimising a JCodec patch. Models that mainly produced explanatory text with minimal code (e.g., CodeLlama, deepseek, StarCoder, WizardCoder, and Zephyr) were excluded due to the difficulty in parsing and limited relevance. StarCoder was also excluded for occasionally outputting non-English responses. Furthermore, we excluded models that either attempted to complete code like adding class Main to the patch (DeepSeek and StarCoder) or included a variety of programming languages beyond Java (Dolphin-mixtral, Magicoder, and WizardCoder). At the time, other candidates like OpenChat and Neural-Chat, similar in size to Mistral , were considered.^5^ OpenChat performed comparably to Mistral (15–30 min run per 10 patches), while Codeup had a worse performance with a 30-60 min run per 10 patches. Hence, due to the rapidly evolving landscape of language models, we selected one of them, Mistral .

To recap, we assessed models based mainly on the quality of their Java code outputs. Yet, we focused on ensuring a diverse representation of models within our evaluation to include models of different sizes: small (TinyDolphin), medium (Mistral) and large (GPT 3.5 Turbo) while considering their performance on CPU for usability. We shared examples from this experiment at Brownlee et al. (2024).

### Experimental environment

We implemented our approach in *Gin*^6^. For the experiments, we installed Java 17 and Maven 3.9.x (managed by SDKMan), as well as Gradle 8, which are pre-requirements for *Gin* and the evaluated projects. We ran our experiments on several servers.^7^. The machine specifications for each experiment are listed in Table 2. For any given project, all local search runs ran on the same machine to ensure that the measured times were comparable among the results for that project.

**Table 2:** Machine specifications for each experiment

Project, search (LLM)	Machine specification
JCodec , RS (OpenAI , Mistral )	AMD Threadripper 3990x, 64C/128T, 128GB, Titan RTX
JCodec , RS (TinyDolphin , Statement)	Intel Xeon W-2245, 8C/16T, 128GB, RTX 2080 TI
JCodec , LS (*)	Intel Xeon 2620v3, 12C/24T, 32GB, Titan X
Gson , RS (OpenAI , Mistral )	AMD Threadripper 3990x, 64C/128T, 128GB, Titan RTX
Gson , RS (TinyDolphin , Statement)	Intel Xeon W-2245, 8C/16T, 128GB, RTX 2080 TI
Gson , LS (*)	Intel Xeon W-2245, 8C/16T, 128GB, RTX 2080 TI
JUnit4 , RS & LS (*)	Intel Xeon 2620v3, 12C/24T, 32GB, Titan X
Commons-Net , RS & LS (*)	Intel Xeon 2620v4, 16C/32T, 32GB
Karate , RS & LS (*)	Intel Xeon 2620v3, 12C/24T, 32GB, Titan X

An experiment is described by the target project, the search type (local search (LS) or random search (RS)) and the LLM model used for LLM edits, or Statement for statement-level edits. An asterisk means the specification refers to all models and edit types.

## Results

In this section, we present and discuss the results of the comparison of statement-level edits and LLM-based edits with different prompting strategies and LLM models. The performance of both a Random Sampling and a Local Search approach is compared using five well-known open-source projects as a benchmark.

### Random sampling

**Fig. 4:**
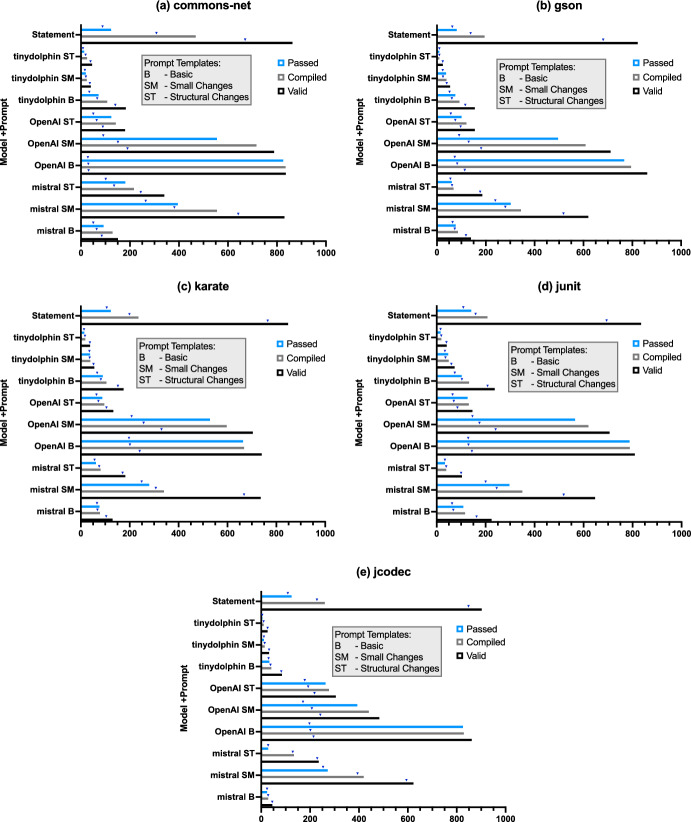
Random Search results for the five projects: Commons-Net , Gson , Karate , JUnit4 and JCodec . Each plot shows the results of a single project for each combination of an LLM and a prompt with the results for the statement-level edits. The number of valid, passed and compiled patches is shown. The triangles indicate the number of unique patches

First, we analyse the statement-level edits and the LLM-based edits for Random Sampling and compare the number of valid patches (successfully parsed), compiling patches, and patches passing all unit tests. Figure 4 shows the achieved results for all studied combinations of the considered prompting strategies and LLM models as well as the results achieved with the statement-level edits for the projects Commons-Net (subplot a), Gson (b), Karate (c), JUnit4 (d), and JCodec (e). The number of unique patches is indicated with a triangle for each configuration.

We observe that most valid patches were found with the statement-level edits on 4 out of 5 projects where always over 800 valid patches were found. For comparison, with the locally executed LLM models TinyDolphin and Mistral , the number of valid patches is always lower, regardless of the prompt used. However, if we analyse the number of found compiling patches and patches passing the unit tests, we see a strong downward trend on all projects with the statement-level edits. On average, less than 50% of the valid patches found with the statement-level edits compiled and less than 20% passed the tests. For the LLM-based edits, the percentage of the found compiling and unit test passing patches is notably higher in relation to the found valid patches. For example, out of 836 valid patches produced by OpenAI with the basic prompt for the Commons-Net project, 835 compiled and 825 passed the unit tests.

**Answer RQ1:** LLMs produce up to 80% valid, compiling, and test passing patches. Therefore, they are effective at producing program variants when prompted to produce an alternative implementation of a given method. While statement-level edits generally find more valid patches, the number of patches that compile and pass the unit tests is higher using LLMs.

Analysing the number of compiling and unit test passing patches, the best results were achieved with OpenAI ’s LLM model with the basic (denoted as B) and the small changes (SM) prompt templates. On average, with OpenAI B about 821 valid, 783 compiling and about 774 passing patches were found. The results achieved with the locally executed LLM models TinyDolphin and Mistral are considerably lower. For example, with Mistral , the best results were achieved with the small changes (SM) prompt with around 402 compiling and 309 passing patches on average. With TinyDolphin the number of found patches passing the unit tests is always lower than 200 regardless of the prompt used. However, we should keep in mind that every call to OpenAI ’s API is billed, while the costs of local LLM models (infrastructure and energy costs) can be adjusted to the user’s needs. In addition, depending on the project, data protection and privacy reasons should also be taken into account, which could favour the use of local or self-hosted LLMs.

**Answer RQ2:** The efficacy of LLMs in producing program variants depends on the model. In general, OpenAI ’s LLM generates more valid, compiling, and test passing program variants when prompted to produce alternative method implementations compared to Mistral and TinyDolphin .

**Answer RQ3:** The best results are achieved with the basic and small changes prompt, depending on the model used. This indicates that in our setup simpler prompts are more effective in generating program variants produced by an LLM when prompted to produce alternative method implementations.

Regarding the number of unique patches found, we have observed that on average the highest number of unique patches was found with the statement-based edits (78–94%). The proportion of unique patches is relatively high for the TinyDolphin (74–100%) and Mistral (56–100%) models. With OpenAI ’s model, on the other hand, unique patches range between 3–79% and often less than 50% of the patches found are unique (especially with OpenAI SM and OpenAI B) indicating the generation of many repeated edits. We measured unique numbers by removing patches with identical strings: it could be interesting to explore how similar the remaining patches are in terms of syntax and semantics in the future.

### Local search

**Fig. 5:**
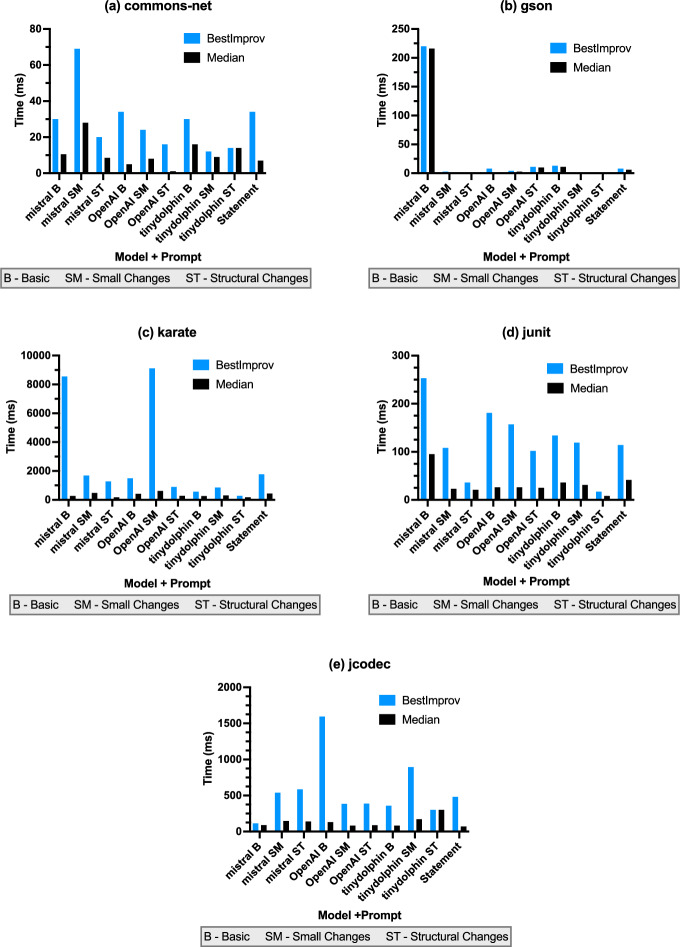
Local Search results for the five projects: Commons-Net , Gson , Karate , JUnit4 and JCodec . Each graph describes the results of a single project for each combination of an LLM and a prompt. The best improvement (bestImprov) and the median improvement (median) are shown in milliseconds

Second, we analyse the statement-level edits and the LLM-based edits for Local Search with a focus on the run time of the found improvements. Figure 5 shows the achieved best as well as the median improvements in milliseconds (ms) for all studied combinations of prompting strategies and LLM models as well as the improvements achieved with the statement-level edits for all considered projects.

For all projects (subplots a-e), we see that the best improvements were found with LLM-based edits. Among these, we see that for three projects the best results were achieved with the Mistral LLM model using the basic (B) prompt or the small changes (SM) prompt, where we observe, e.g., best improvements for the projects Commons-Net (a), Gson (b), and JUnit4 (d) of 69 ms, 220 ms, and 253 ms, respectively. The overall best improvements for Karate (c) and JCodec (e) were found with OpenAI of 9109 ms, and 1595 ms, respectively. Looking at the median improvement, the best results for Commons-Net (a), Gson (b), and JUnit4 (d) were found with Mistral again. According to the median improvement, OpenAI performed best for Karate (c) and TinyDolphin for JCodec (e).

**Answer RQ4:** For all studied projects, the best runtime improvement is found with an LLM-based edit. Moreover, higher median runtime improvements were found with LLM-based edits compared to statement edits. However, there are large differences depending on the model and prompt.

Following RQ4-Answer, where LLM-based edits led to the best runtime improvements, one might assume that a better prompt or sequence of prompts is required (e.g. to address the low validity rate of LLM-generated code (Zhang et al. 2023; Xia et al. 2024)). This seems promising, worth sacrificing *Gin* ’s performances as LLMs can detect and fix programs through few-shot prompting (Jin et al. 2023; Albuquerque et al. 2024). However, a patch that fails to compile likely indicates deeper issues and poor quality. We have observed that LLMs often apply examples as-is without generalising them to code snippets being edited (e.g. adding a statement from an example as-is instead of applying an edit in a similar way as presented in the example). We discuss this in the *Invalid Code* paragraph in the next section (subsection 5.3).

Fixing such patches is unlikely to result in valid functional code that passes the unit tests (Pearce et al. 2022). Nevertheless, a more complex mechanism is likely necessary for a few-shot prompting to be effective in patch editing and passing the unit tests. We leave it for future work.

There is, of course, additional time overhead when using the LLMs to generate edits. Table 3 reports the relative wall clock times for the Local Search runs with each model on each project. Raw times for these runs are provided in the artefact (Brownlee et al. 2024). The times were measured from the beginning to the end of the sampling run, and include all API calls, LLM response times, parsing, compilation and testing of patches, and logging activity. The numbers reported in the table are computed as the ratio of the runs using LLMs vs classic GI statement edits so, for example, the run on Commons-Net with OpenAI was 7.88 times as long as the run on Commons-Net using statement edits. The run with Mistral was 91.92 times as long as the run using statement edits. In concrete terms, the run exploring 1000 statement edits completed in 818 seconds, with OpenAI’s run taking 6449 seconds and Mistral's taking 75187 seconds. Put another way: in the same time it took to generate and evaluate 1000 statement edits, it was only possible to generate and evaluate 127 OpenAI edits or 11 Mistral edits. There are several confounding factors to these numbers: as well as all the usual noise in measuring run times (even given dedicated machines), the Ollama bug noted in Sect. 5.3 and variations in OpenAI’s server response times make any precise comparisons impossible. In this light the figures are reported only to provide some context. For some of the projects, testing the patches took long enough that the extra overhead to call the LLM was lower in impact than might be expected. For the OpenAI sourced edits, the Local Search took around 1–3.6x as long as statement edits with JUnit4 and Karate. Given that OpenAI edits passed the unit tests more than 4x as often with each of these projects, the additional time taken by the LLM is of value. For the other projects, while the extra time taken to run the models does not lead to a proportionate increase in test-passing variants generated, LLM edits did produce larger speedups than statement edits. Assuming the improved application will be run many times, the one-off time investment in the optimisation stage is worthwhile.

**Table 3:** Relative run times for the local search experiment for each model on each of the target projects

	**Project**				
Model	Commons-Net	Gson	jcodec	JUnit4	Karate
Mistral	91.92	19.21	83.25	21.59	3.73
OpenAI	7.88	24.86	28.03	3.64	1.05
TinyDolphin	61.05	13.92	21.62	12.77	1.02

The numbers reflect the wall-clock time, relative to the equivalent for the statement edits, for the complete sampling run including calls to the model, as well as parsing, compilation, and testing of the patches.

**Answer RQ5:** In our setup, either OpenAI or TinyDolphin have the lowest runtime overhead compared to statement edits.

### Qualitative analysis

We present here an error analysis to explain why some LLM-generated edits fail to compile or pass tests. Our logs (stderr) are available at Brownlee et al. (2024). However, understanding the errors beyond the common reasons identified in this section requires a thorough familiarity with each project.

Certain combinations of models and prompts outperformed others. We conducted a manual analysis of the logs, examining the responses from LLMs and *Gin* ’s parsing of the suggested variants to understand better the source of failures. Through this process, we categorised these into three categories: Cases where (1) no replacement was found when parsing the response, (2) a replacement was offered but failed compilation or was invalid according to regression tests, and (3) a replacement was found, successfully compiled, and passed all regression tests, yet offered no advantage. We shared the logs analysed in this section as part of our artefact(Brownlee et al. 2024).

**A Bug in the** *Ollama***Service. ** The *Ollama* service halted unexpectedly during the experiment. We noticed it happened more frequently with TinyDolphin compared to Mistral . It is a known bug of *Ollama* previously reported.^8^ We attempted to mitigate this by pulling specific versions of *Ollama* known to be less prone to the problem, but we observed no difference. We opted to implement a simple mechanism to automatically restart the *Ollama* service during *Gin* ’s execution using a script available as part of our artefact (Brownlee et al. 2024), which led to some responses being lost.

**Issue with Code Formatting. ** While parsing code with *Gin* , we developed a basic parser to detect code notations (```<CODE>```) with or without a java label. However, all three models displayed creativity in marking sections as code, which posed challenges for our parser in precisely identifying the $\texttt {<COUNTER>}$ variants requested from the model. Some examples:

1. No use of code notations, marking it as just regular text;
2. TinyDolphin and Mistral returned code that is not written in Java (e.g. Python, Ruby, PhP, or Kotlin) and tagged it accordingly (e.g. Python:<line-break>```python);
3. Applied non-consistent ways of marking the code as Java code, varied even within the same execution of *Gin* , such as Java<line-break>```<CODE>```, java<line-break>```java<CODE>``` or ```java java<line-break><CODE>``` (with java tag having some variations such as JAVA, java:, Java:, etc.); and
4. Used plenty of free text, making it difficult to parse, like <free-text> <line-break>```java<CODE>``` with some variations of free text before the code such as (Strict Implementation), implementation, Java implementation 1:, (With ‘dfs‘ check), Java implementation #1:, With ‘dfs‘ check and ‘SimpleDateFormat‘ instantiation outside the if condition:<line-break><line-break>.

**LLM Response Contains No Code.** Some prompts’ responses from TinyDolphin and Mistral did not contain any code, irrespective of the prompt type. Some of them declared the task is impossible or done, e.g. with TinyDolphin for this code in the prompt ```{ sb.append("return ");}```, the LLM returned the following response:
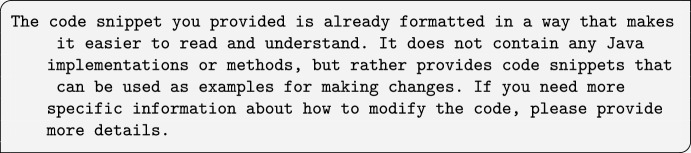


Other responses contained pseudocode or a list of suggestions as free-text, e.g., with Mistral and the following code in the prompt ```{ rhs = "";}```, the model’s response was:
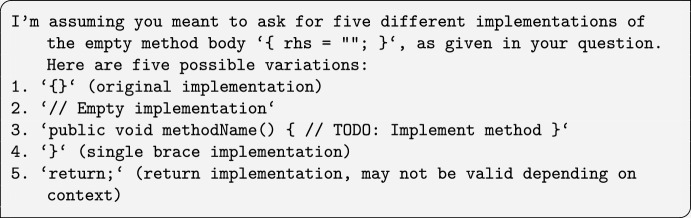


Note that OpenAI had a similar kind of response, but it made sure to surround each code edit suggestion in code notation as a comment, like this: ```{ // implementation using Java 8 Streams}```.

Last, LLMs have explanatory capabilities, which can lead to the following response (Mistral and the small changes prompt):
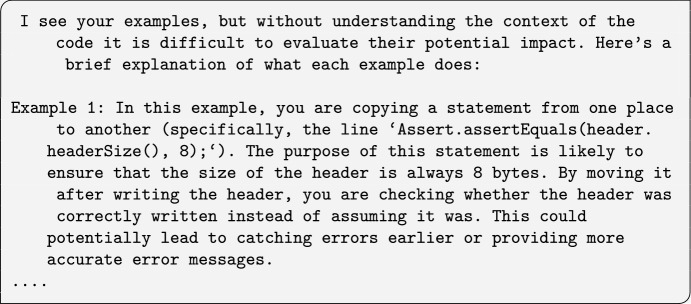


In this example, Mistral explained each of the code examples in Fig. 2 when presented relatively long code snippet to patch.

**Invalid Code. ** The LLM’s response included an invalid code. It could be due to various reasons like undeclared variables or syntax errors. However, we identified several reasons specific to LLMs that can lead to invalid code.

OpenAI with structural changes attempted to turn the following code snippet ```{ for (DateFormat dateFormat: dateFormats) { try { return dateFormat.parse(s); } catch (ParseException ignored) } }``` into a method, abandoning completely the original task of seeking replacements for it, returning this method with some headers:
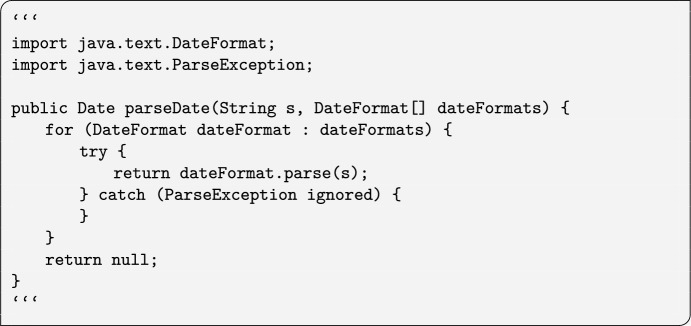


as the original code was part of a block of instructions in a method, embedding the response suggestions led to a compilation failure. We observed this also in Mistral and TinyDolphin ; TinyDolphin (Commons-Net, Random Sampling, basic prompt) identified the origin of the code snippet from Commons-Net ’s GitHub project, returning the entire Java implementation class as the response.

For all three models when using small changes or structural changes prompts, the response sometimes contained the code examples in Fig. 2 and Fig. 3, respectively. For example when prompting with this code snippet: ```{sb.append("return ");}```, OpenAI with Fig. 3 was:
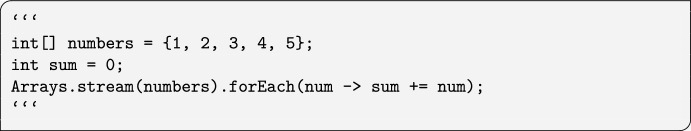


that would likely lead to test suite failure; specifically in this case, *Gin* failed much earlier because the LLM had appended free text too, resulting in the response not being parsed properly by *Gin* .

**Useless Edits. ** When mutating code snippets, LLM occasionally suggests useless edits. During our analysis, we found some of these: (1) No actual edit: The response remained identical to the prompt; (2) Comment-only edits: Edits were limited to the code’s comments; (3) Explaining the Code: LLM added comments to explain the code, but these additions don’t improve performance; and (4) Repetitive responses: The same response was repeated multiple times, offering no new insights.

## Threats to validity

In this work, we used different LLMs to investigate their suitability for code mutations in the well-known GI system *Gin* . However, common threats with LLMs are that they behave like a black box for their users and that newer versions may produce a different output, which could influence the reproducibility of the results. To mitigate these risks, we not only used OpenAI ’s GPT 3.5 Turbo via API, as this service could change at any time but also the TinyDolphin and Mistral models, which can be installed locally. Consequently, we specified the versions used for TinyDolphin and Mistral . Compounding this issue, even with temperature set to zero, LLMs show non-determinism (Ouyang et al., 2023). While this variability may actually be beneficial for search, adding diversity (Blyth et al., 2024), it nevertheless makes the replication of the results for a study like ours difficult, so the best that can be done is to provide the logs tracking the LLM responses as we have done in our artefact. Future study of techniques to minimise this variability such as temperature tuning or consistency checks would definitely be interesting to pursue.

Moreover, the number of software projects and LLM models studied in our experiments is limited, which could have biased the drawn conclusions. However, compared to our preliminary work, we have significantly increased the number of studied projects and LLM models and could confirm previous findings and gain new insights. We considered projects of up to 132k significant lines of code: enterprise systems can be significantly larger and it is not clear how far the approaches will scale. We expect that as the target application's size increases, larger context windows for the LLM interactions will be required, the search space of possible edits will increase exponentially, and testing will become more time-consuming. It is reasonable to think that these issues can be overcome: LLM context windows increase with each new development, and existing GI success stories suggest that search algorithms scale well to industrial scale software with the traditional operators.

In addition, the run time of the improvements is difficult to measure precisely. Furthermore, we assumed the validity of the test suites in our experiments. However, both risks affect most of SBSE research. To counteract them, we carried out our measurements in accordance with the GI literature.

## Related work

The last few years have seen a rapid uptake of large language models (LLMs) to solve software engineering problems (Fan et al. 2023) such as program repair (Zhang et al. 2024). Research in the area has resulted in many LLM-based end-to-end tools that address the functional improvement of software (Bouzenia et al. 2024; Xia et al. 2023; Jin et al. 2023).

Aside from end-to-end solutions to software engineering tasks, LLMs have also been augmented into existing ones and used as optimizers (Hemberg et al. 2024). One such example is the use of LLMs for improving the search in existing search-based approaches such as search-based test generation (Lemieux et al. 2023) and fault localization (Murtaza et al. 2024).

Since the emergence of genetic improvement research, the canonical edits that delete, swap, replace, or insert pieces of code (statements, lines of code, assembly instructions etc.) have been used. Given the large search space for application of such mutation operators, more specialised ones have been introduced, such as insertion of a break/return (Brownlee et al. 2020), and others., In our preliminary work, we investigate whether taking this a step further by prompting LLMs for code mutations could be a viable approach in the GI context (Brownlee et al. 2024). Similarly, Hu et al. (2023) used ChatGPT to generate variations to the seed program for a greybox fuzzer and Birna et al. (2024) use LLMs as a recombination operator for GI. It is worth noting that most of the previous work focused on the power of commercial LLMs, such as GPT. This provides a restriction on the availability of such models (both in terms of access and computing resources required). Here we want to see how well smaller, open-source models perform.

Furthermore, we explore different prompts. The field of prompt engineering deals with crafting prompts to gain more successful results from LLMs, yet the field does not yet provide clear guidelines, beyond those specific to each model. In this area, Chain-of-Thought prompting gained some attention (Wei et al. 2022). The method divides a given task into subtasks, for which prompts are individually crafted. It is yet unclear which prompts should be used for a given task, such as mutation operator generation. There have been a few attempts to automate this process though. Fernando et al. (2024) proposed to evolve mutations that change the prompts themselves, guided by a fitness function specific to a given task. Guo et al. (2023) proposed to evolve a population of prompts using hand-crafted mutations and crossover operators. While the second approach is freely available it is extremely time-consuming and it is unclear whether the operators would yield better results in our case. Nevertheless, use of such tooling could be used in future work.

## Conclusions and future work

Genetic improvement of software is highly dependent on the mutation operators it utilises in the search process. To diversify the operators and enrich the search space further, we previously incorporated a Large Language Model (LLM) as an operator. The present paper builds on that work to explore three models and three prompting strategies on five software programs.

We found that, although more valid and diverse patches were found with standard edits using Random Sampling, up to 9 times more patches passing the unit tests were found with LLM-based edits. Simpler prompts appeared to generate test-passing edits more often. The larger models of OpenAI and Mistral appeared to be more successful than TinyDolphin . For example, with OpenAI ’s LLM and the basic prompt around 774 passing patches were found on average (compared to around 118 passing patches found with statement edits on average). However, the statement edits still produced more unique variations to the code, so there is potential in exploring approaches combining both LLM and ‘classic’ GI edits. It would be also interesting to carry out a deeper analysis of which kind of edits works best and where — perhaps exploring what features of the target source code make it more amenable to improvement.

Our preliminary exploration (Brownlee et al. 2024a) hinted that the statement edits found the best runtime improvement. The additional prompts and models we analysed in the present work led to the best runtime improvements being found with LLMs for all 5 target projects. The improvements found with LLMs were between 2 and 27 times greater than those found with statement edits.

Our experiments revealed that the prompts used for LLM requests greatly affect the results but there is clearly still much to be explored in terms of models and prompt strategies. A systematic exploration of different combinations of prompting elements would be a very interesting direction to pursue. This could include variations we have not yet considered, such as the inclusion of the optimisation objectives (“reduce runtime”) in the prompt, or including feedback about errors in the suggested patches. It might also be helpful to mix prompts: e.g., starting with the basic prompt then switching to the small changes prompt to make larger edits that break out of local minima. Further, the possibility of combining LLM edits with others such as standard copy/delete/replace/swap or PAR templates (Kim et al. 2013) could be interesting.

## Data Availability

Experimental data can be found in our Zenodo artefact (Brownlee et al. 2024). The code can also be found on GitHub https://github.com/gintool/gin, llm branch, commit 9fe9bdf.

## Footnotes

1 https://javaparser.org

2 Since all our benchmarks use Maven, we use Maven to run tests.

3 https://github.com/langchain4j/langchain4j

4 https://github.com/amithkoujalgi/ollama4j

5 Most language models are of medium size due to their balanced performance and resource demands.

6 GitHub https://github.com/gintool/gin, llm branch, commit 9fe9bdf.

7 The environments of all machines are listed in Brownlee et al. (2024)

8 E.g. https://github.com/ollama/ollama/issues/1863.
